# Optimization of a Capacitated Vehicle Routing Problem for Sustainable Municipal Solid Waste Collection Management Using the PSO-TS Algorithm

**DOI:** 10.3390/ijerph17062163

**Published:** 2020-03-24

**Authors:** Qingqing Qiao, Fengming Tao, Hailin Wu, Xuewei Yu, Mengjun Zhang

**Affiliations:** 1 College of Mechanical Engineering, Chongqing University, Chongqing 400044, China; qiaoqingqing@cqu.edu.cn (Q.Q.); wu_hailin@foxmail.com (H.W.); yuxuewei@cqu.edu.cn (X.Y.); zhangmengjun@cqu.edu.cn (M.Z.); 2 School of Management Science and Real Estate, Chongqing University, Chongqing 400044, China

**Keywords:** municipal solid waste collection, sustainable management, capacitated vehicle routing problem, two-phase algorithm

## Abstract

Sustainable management of municipal solid waste (MSW) collection has been of increasing concern in terms of its economic, environmental, and social impacts in recent years. Current literature frequently studies economic and environmental dimensions, but rarely focuses on social aspects, let alone an analysis of the combination of the three abovementioned aspects. This paper considers the three benefits simultaneously, aiming at facilitating decision-making for a comprehensive solution to the capacitated vehicle routing problem in the MSW collection system, where the number and location of vehicles, depots, and disposal facilities are predetermined beforehand. Besides the traditional concerns of economic costs, this paper considers environmental issues correlated to the carbon emissions generated from burning fossil fuels, and evaluates social benefits by penalty costs which are derived from imbalanced trip assignments for disposal facilities. Then, the optimization model is proposed to minimize system costs composed of fixed costs of vehicles, fuel consumption costs, carbon emissions costs, and penalty costs. Two meta-heuristic algorithms, particle swarm optimization (PSO) and tabu search (TS), are adopted for a two-phase algorithm to obtain an efficient solution for the proposed model. A balanced solution is acquired and the results suggest a compromise between economic, environmental, and social benefits.

## 1. Introduction

With the rapid development of the economy, China is confronted with great challenges with respect to waste management due to mass production and consumption. Furthermore, increasing urbanization makes municipal solid waste (MSW) management more complicated due to the large populations in cities. MSW is defined as the most complicated form of solid waste, including waste from residential, commercial, and institutional sources, and does not include industrial, construction, and hazardous waste [1,2,3]. The management of MSW generally includes collection, transportation, processing, and disposal of waste [4]. Among these services, approximately 75–80% of the solid waste management budget is spent on collection and transportation costs [5]. Therefore, even a small improvement in waste collection can greatly reduce cost. In addition, sustainability is an increasing societal concern nowadays, requiring an active organizational approach [6]. Within such a context, MSW collection management organizations face big challenges with respect to promoting sustainable development of planning and operation of the collection process. 

The common perception around the world is that sustainable development has to embrace three fundamental sustainability pillars: economic growth, environmental protection, and social equity [7]. In order to address the challenges of sustainable development, organizations should effectively manage the collection process by considering economic, environmental, and social benefits simultaneously. Therefore, this paper utilizes three dimensions—economic, environmental, and social—to evaluate the sustainability of the MSW collection process. 

Carbon dioxide emissions are the most common assessment criterion of the environmental dimension in MSW collection [8]. Transportation, which is an integral element of the MSW collection process, is a crucial sector of energy consumption and a key driving force of carbon dioxide emissions in China [1,9]. The resulting carbon emissions have significant effects on public health and global warming both directly and indirectly [9]. In 2005, Chinese government made a promise to reduce carbon dioxide emissions per unit of GDP by 40%–45% by 2020 [10]. Thus, in order to make MSW collection more environmentally friendly, this paper considers carbon dioxide emissions generated from transportation, since CO_2_ is the main source of greenhouse gases (GHGs) [11]. 

In addition to the consideration of the environmental component, the social benefits of MSW collection are also tackled in this paper. For most previous studies in MSW collection, lower operating costs and carbon emissions have been considered, but social sustainability is rarely mentioned [6,12]. After collecting a certain amount of waste, vehicles need to unload the waste at the recycling facilities. However, even if a schedule has low operating and fixed truck costs, it cannot be used unless a balanced assignment of collection trips to recycling facilities is attained [13]. If there are long queues at disposal facilities, greater costs and fuel consumption will result. Meanwhile, an unbalanced allocation of vehicles would also cause other waste disposal facilities to be in an idle state without any mission to undertake. Such a wide discrepancy of the workload has a negative effect on equity perceptions, which are beneficial for retaining employees in the organization [14,15]. Therefore, this paper aims at balancing the workload of each disposal facility, so as to reduce unnecessary fuel consumption and promote social equity.

Sustainable development is a multi-dimensional concept which emphasizes integration and the striking of a dynamic balance between economic, social, and environmental aspects [16]. As to the requirements of sustainable development, this paper aims to facilitate decision-making for the MSW collection system in order to make the collection process more economical, more environmentally friendly, and more socially oriented. This paper is a development study of previous research done by Li et al. [13] in order to cover research gaps such as the consideration of vehicle capacity and carbon emissions. We demonstrate that our approach could result in a preferable balance among the economic and environmental benefits as well as social equity. 

The rest of the paper is organized as follows. Section 2 presents a literature review of related work and the existing research gap. Section 3 proposes the mathematical model. Section 4 describes the proposed algorithms. In Section 5, the algorithm experiment and model experiment are presented. The discussions and future research directions are discussed in Section 6, while conclusions are in Section 7.

## 2. Literature Review

The main purpose of this paper is to obtain an optimal distribution plan with consideration of sustainable development in MSW collection. A capacitated vehicle routing problem (CVRP) model is developed for the MSW collection optimization problem. Therefore, the literature review is composed of two parts: research on sustainable development in MSW collection and research on algorithms for the CVRP model.

### 2.1. Research on Sustainable Development in MSW Collection

Increased environmental awareness and human welfare consciousness from public, sustainable development has emerged with respect to MSW collection management systems. For economic feasibility, environmental benefits, and social justice, reasonable balance and adjustments are needed for designing the operational MSW collection system [9].

In recent years, there has been a considerable increase in the number of studies taking into account environmental issues by minimizing GHG emissions from logistic networks [17,18,19]. The majority of the published research consider economic costs and environmental benefits together in the MSW collection process. Mahmuda Akhtar et al. [17] introduced a concept of TWL (threshold waste level) to increase waste collection efficiency and reduce economic and environmental costs, including fuel consumption, fuel costs, and CO_2_ emissions. In their follow-up study, this method had an excellent optimization effect for week-long scheduling [18]. By using multi-objective decision-making approaches, comprehensive optimization was often performed so as to keep a balance between various objectives [9]. Tamás Bányai et al. [20] introduced a cyber-physical system to simulate the waste collection process of downtown areas and guarantee energy usage cost-efficiency and environmental awareness of GHG emissions simultaneously. Bektas and Laporte [19] put forward the pollution-routing problem, and designed vehicle routes aimed at minimizing the fixed costs of drivers, fuel consumption, and CO_2_ emission costs. Maurizio Faccioa et al. [21] proposed a comprehensive multi-objective VRP (vehicle routing problem) model aimed at minimizing the total covered distance, the necessary number of vehicles, and the environmental impact.

Environmental aspects have been given attention in prior literature, but social concerns have rarely been studied [12]. Vinay Yadav et al. [22] aimed at sustainable collection and transportation of MSW, and classified the corresponding mathematical models into three domains: vehicle routing, facility location, and flow allocation (efficient allocation of the MSW stream to processing facilities). Heidari et al. [2] incorporated the social criterion so as to maximize the job opportunities brought about from the establishment of new disposal facilities in the MSW management system. Ramos and Oliveira [23] studied the performance of equity with multiple depots in a recyclable waste collection system. By defining the service areas of the depots, the difference in workload (working hours) among depots was minimized. Li et al. [13] considered the social benefits of balancing the collection trips assigned to recycling facilities. In this way, all facilities received almost the same number of the tasks so as to guarantee the employment of deprived people. Equity not only exists in the collection routes assigned for disposal facilities, but also can be addressed through minimizing the maximum route length or the route length gap between the longest and the shortest route lengths [6]. Jozefowiez et al. [24] and Reiter and Gutjahr [25] studied the vehicle routing problem with route balancing (VRPRB) and constructed a bi-objective model in order to minimize travel cost (or route length) and the maximum route length.

In China, government and scholars have begun to pay attention to sustainable development of MSW management. In response to mounting environmental issues surrounding food waste disposal, the Chinese National Development and Reform Commission approved 100 pilot cities to implement kitchen waste disposal projects with government investment to encourage waste classification from 2010 onwards [26]. Chu et al. [27] studied policy-making trends in the area of municipal solid waste for further sustainable development in China. Ma et al. [28] focused on the source of MSW and studied the factors that influences sorting collection behavior in the developing areas in China. Wang et al. [29] quantitatively evaluated economic and environmental performance of an integrated MSW treatment center in Inner Mongolia province, China. Lu et al. [30] searched for the most cost-effective and environmentally benign solutions for an MSW collection scheme system in Shenzhen, China.

From the research on sustainable development on MSW collection above, we see that these studies are aimed at achieving at the lowest cost and levels of carbon emissions, with little attention given to social benefits. In China in particular, research on the sorting and treatment of MSW has been given sufficient attention; however, only limited literature focuses on the sustainable development of MSW collection. Suocheng et al. [31] pointed out that the MSW ‘industry chain’ of sorting, recycling, collection, transportation, and treatment should be emphasized as an engine for economic growth in China. Therefore, for the problem of MSW collection, this paper assumes that if vehicles routes are of lower cost, result in fewer emissions, and are more focused on equity, collection can be sustainable. This paper defines equity as balanced trip assignments of disposal facilities so as to make sure that each facility receives roughly the same number of tasks.

### 2.2. Research about Algorithms for the CVRP Model

The literature has increasingly considered the vehicle capacity constraint in the VRP, denominated CVRP [32]. The relevant mathematical models of the CVRP and optimization algorithms for MSW collection have been continuously extended and developed. Rodriguez-Martin et al. [33] presented an integer linear programming formulation for the periodic capacitated vehicle routing problem (PCVRP) and solved it by an exact branch-and-cut algorithm. Benrahou et al. [34] adopted a heuristic algorithm called the nearest insertion algorithm (NIA) in the CVRP model to reduce collection distance and compare the solution effectiveness of the heuristics with the current method. Vera Hemmelmayr et al. [35] developed an efficient hybrid solution method based on variable neighborhood search (VNS) and dynamic programming for the periodic vehicle routing problem with intermediate facilities (PVRP-IF). They found that a sophisticated insertion procedure can improve the solution quality if it is combined with a local search algorithm. Pelletier et al. [32] proposed a two-phase heuristic method based on large neighborhood search and identified new best solutions for robust CVRP.

However, heuristic algorithms lack precision and require a long execution time in collecting solid waste [36]. Thus, meta-heuristic approaches have become popular in recent years because these techniques provide a sufficiently good solution for collection optimization even when incomplete information or limited computation capacity is given [18]. Some popular meta-heuristic approaches include ant colony optimization (ACO) [37], the genetic algorithm (GA) [38], and particle swarm optimization (PSO) [18,38]. Liu and He [37] adopted a clustering-based multiple ant colony system approach called the CMACS algorithm in vehicle routing problems with time windows and intermediate facilities (CVRP-IF) to improve the route compactness. The robustness of the proposed algorithm was also tested by conducting two standard instances. The PSO is simple and flexible, but usually obtains the local optimum [11]. Hence, some papers incorporated other meta-heuristic algorithms so as to avoid roundabout searching. Kuo et al. [38] combined hybrid particle swarm optimization (HPSO) with genetic algorithm (GA) in a method known as HPSOGA for solving capacitated vehicle routing problems with fuzzy demand (CVRPFD). They verified the proposed method by using some CVRPFD datasets which were modified from CVRP instances and applied it to a real garbage collection system. Yangkun et al. [39] constructed a double-objective CVRP model to save the costs of low-carbon logistics and reduced carbon emissions together. By employing the corresponding adaptive tabu search (TS) algorithm, the global optimization ability of the proposed algorithm is enhanced. 

In summary, for sustainable development in MSW collection, there is still little research that considers the three factors of economy, environment, and society simultaneously; for algorithms used in the CVRP model, previous studies rarely adopt hybrid meta-heuristic algorithms which can learn from each other and mutually progress. Considering the key characteristics and gaps of the learned literature, this paper adopts two meta-heuristic algorithms, the PSO and TS, and focuses on maximizing the comprehensive benefits which are composed of economic, environmental, and social benefits. The economic benefit has two parts: the fixed cost for the staff of vehicles and fuel consumption costs. The environmental factor is assessed through the CO_2_ emissions from transportation activities and the emissions are transformed to the costs by per unit carbon cost. The social factor is considered by defining a balanced solution in terms of the number of trips received among disposal facilities.

## 3. Mathematical Model

### 3.1. Problem Description

**Figure 1:**
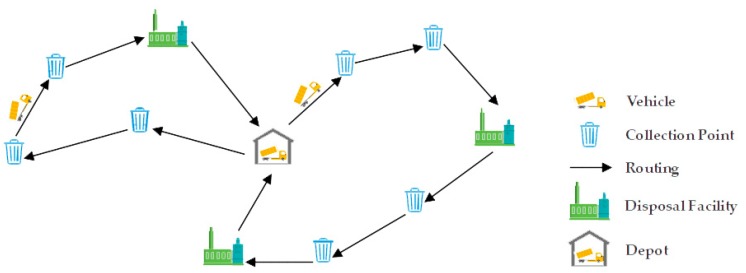
Simplified routing diagram of municipal solid waste (MSW) collection.

In the municipal solid waste (MSW) collection problem, the CVRP could be modeled as a minimum-cost flow problem in order to fit various requirements. It can be defined as collecting waste from a set of collection points by a homogenous or heterogeneous fleet of vehicles of fixed capacity that cannot be violated, each starting from and returning to the depot [17]. After collecting a certain amount of waste, vehicles need to unload at a disposal facility because of the capacity constraint of vehicles. However, due to the processing limit of disposal facilities, it is necessary to balance workload of disposal facilities. This paper adopts the penalty costs introduced by Jing-Quan Li et al. [13]. When the number of assignments distributed to a facility exceeds a given limit, it imposes a penalty cost in the total costs so as to diminish the unbalanced condition. After visiting a disposal facility, the empty vehicles can continue their trip to collect more waste. The objective of this model is to minimize total costs so as to arrange collection routes and distribute an appropriate number of vehicles to disposal facilities. Referring to Banyai et al. [20] and Kim et al. [40], a simplified diagram of the MSW collection process is presented in Figure 1. 

### 3.2. Problem Assumptions

(1) Only one depot is considered in this model. All vehicles start from the depot at the same time, and return there eventually.
(2) The vehicles start and end their trips with an empty load.
(3) All vehicles are homogeneous with the same capacity limit.
(4) The collection points are also homogeneous with the same capacity limit. Each point should be served once by one vehicle.
(5) The vehicles may take multiple trips.

### 3.3. Parameters and Variables

Table 1 presents the corresponding parameters and variables in this model. 

### 3.4. Model Construction

#### 3.4.1. Objectives Function

(1) Vehicles’ Fixed Costs

In waste collection management, each vehicle has at least a driver and a crew. Thus, fixed costs for drivers’ and crews’ salaries need to be considered in the model. In this paper, the vehicles’ fixed costs in the CVRP model can be expressed as Equation (1).

(1) $$C_{1}=c_{v}\overset{H}{\underset{h=1}{\sum }}\overset{N+S}{\underset{i=0}{\sum }}\overset{N+S}{\underset{j=0}{\sum }}x_{\mathrm{ijh}}$$

(2) Fuel Consumption Costs

Fuel consumption of vehicles is affected by many factors, such as car characteristics, road condition, running speed, etc. It is difficult to take all factors into consideration. As per Xiao et al. [41], this paper calculates fuel consumption $F_{fuel}$ and its costs $C_{2}$ as Equations (2) and (3).

(2) $$F_{\mathrm{fuel}}=\overset{H}{\underset{h=1}{\sum }}\overset{N+S}{\underset{i=0}{\sum }}\overset{N+S}{\underset{j=0}{\sum }}\overset{M}{\underset{m=1}{\sum }}\overset{S}{\underset{r=1}{\sum }}\left(\eta_{0}+\frac{\eta -\eta_{0}}{Q}Q_{\mathrm{ijh}}\right)d_{\mathrm{ij}}x_{\mathrm{ijh}}z_{\mathrm{mhi}}^{r}$$

(3) $$C_{2}=c_{f}F_{\mathrm{fuel}}$$

(3) Carbon Emission Costs

The carbon emissions of transportation are proportional to fuel consumption. This paper refers to the literature [42] to calculate the amount of carbon dioxide from fuel consumption. Therefore, the carbon emissions $E_{co_{2}}$ and its costs $C_{3}$ of the CVRP model can be expressed as Equations (4) and (5).

(4) $$E_{{\mathrm{co}}_{2}}={\lambda F}_{\mathrm{fuel}}$$

(5) $$C_{3}=c_{e}E_{{\mathrm{co}}_{2}}$$

(4) Penalty Costs

When the number of vehicles assigned to a disposal facility surpasses its processing capacity, penalty costs are imposed on the facility. The given limit of each disposal facility is related to the capacity of all disposal facilities and the total number of collection trips. The given capacity limit $U_{r}$ and penalty value *p* are determined by computational tests. Referring to Jing-Quan Li et al. [13], this paper calculates penalty costs $C_{4}$ as Equation (6).

(6) $$C_{4}=p\overset{M}{\underset{m=1}{\sum }}\overset{S}{\underset{r=1}{\sum }}f_{m}^{r}$$

#### 3.4.2. Model Setting

Based on the detailed analysis of four optimization objectives, the mathematical model of the CVRP is shown as follows:

(7) $$\mathrm{Min}\;F=c_{v}\overset{H}{\underset{h=1}{\sum }}\overset{N+S}{\underset{i=0}{\sum }}\overset{N+S}{\underset{j=0}{\sum }}x_{\mathrm{ijh}}+\;\left(c_{f}+c_{e}\lambda \right)\overset{H}{\underset{h=1}{\sum }}\overset{N+S}{\underset{i=0}{\sum }}\overset{N+S}{\underset{j=0}{\sum }}\overset{M}{\underset{m=1}{\sum }}\overset{S}{\underset{r=1}{\sum }}\left(\eta_{0}+\frac{\eta -\eta_{0}}{Q}Q_{\mathrm{ijh}}\right)d_{\mathrm{ij}}x_{\mathrm{ijh}}z_{\mathrm{mhi}}^{r}+p\overset{M}{\underset{m=1}{\sum }}\overset{S}{\underset{r=1}{\sum }}f_{m}^{r}$$

Subject to:

(8) $$\overset{N}{\underset{j=1}{\sum }}Q_{0\mathrm{jh}}=0,\;\forall h\in K$$

(9) $$\overset{N}{\underset{i=1}{\sum }}Q_{\mathrm{ijh}}\leq Q,\;\forall h\in K$$

(10) $$\overset{H}{\underset{h=1}{\sum }}y_{\mathrm{ih}}=1,\;\forall i=1,\;2,\;\ldots ,\;N$$

(11) $$\overset{N}{\underset{i=0}{\sum }}\overset{H}{\underset{h=1}{\sum }}x_{\mathrm{ijh}}=1,\;\forall j=1,\;2,\;\ldots ,\;N$$

(12) $$\overset{N}{\underset{i=0}{\sum }}\overset{H}{\underset{h=1}{\sum }}Q_{\mathrm{ijh}}-\overset{N}{\underset{i=0}{\sum }}\overset{H}{\underset{h=1}{\sum }}Q_{\mathrm{jih}}=q_{j},\;\forall j=1,\;2,\;\ldots ,\;N$$

(13) $$x_{\mathrm{rjh}}\leq z_{\mathrm{mhi}}^{r},\;\forall h\in K,\;r\in R,\;m\in T,\;i,\;j=0,\;1,\;\ldots ,\;N+S$$

(14) $$\overset{N}{\underset{i=0}{\sum }}\overset{H}{\underset{h=1}{\sum }}x_{i0h}=1$$

(15) $$d_{\mathrm{ij}}=d_{\mathrm{ji}},\;\forall i,j=0,\;1,\;2,\;\ldots ,\;N+S$$

Equation (7) indicates that the goal of the model is to minimize the total costs of waste collection management, including fixed costs of vehicles, fuel consumption costs, carbon emission costs, and penalty costs. 

Constraint (8) ensures that all vehicles start from the depot with empty load. Constraint (9) ensures that the load of each vehicle will not exceed its own capacity. Constraints (10) and (11) illustrate that all points are served and each point is visited once by one vehicle. Constraint (12) addresses that the vehicle must empty the collection point visited. Constraint (13) states that only when vehicle *h* visits disposal facility *r* can the facility be used for dumping waste. Constraint (14) ensures that all vehicles return to the depot eventually. Constraint (15) ensures that the distance between two points is the same in both directions.

## 4. Algorithm Description

The CVRP model is built based on the present MSW collection problem. Two kinds of meta-heuristic algorithms, PSO and TS, are embedded in this model to solve the routing and scheduling optimization problem. Since the PSO algorithm usually falls into local optimum, combining with TS can avoid roundabout searching [11] and decrease the probability of premature phenomena. The proposed PSO-TS algorithm includes two phases. In the first phase, the PSO algorithm is applied to obtain an initial optimal solution. In the second phase, the TS algorithm is adopted to optimize the initial optimal solution generated by PSO. The notations and parameters of PSO-TS algorithm are listed in Appendix A Table A1.

### 4.1. Algorithm Step Design

Step 1: Initialization.

(a) The length of particle code *VarSize*, the number of population *nPop*, and maximum number of iterations *MaxIt* are initialized.
(b) PSO parameters are set: maximum value of inertia weight *w_max_*, minimum value of inertia weight *w_min_*, variance of random inertia weight σ, random value of *R*_1_, *R*_2_, and acceleration factors *C*_1_, *C*_2_.
(c) TS parameters are set: tabu length TL, neighborhood size NS, and candidate size CS.
(d) For each particle, initial position *X_i_* and velocity *V_i_* are determined as per Equation (16).

(16) $$\left\{ \begin{array}{c} V_{i}=\mathrm{rand}(\mathrm{VarSize})*(\mathrm{VelMax}-\mathrm{VelMin})+\mathrm{VarMin},\\ X_{i}=\mathrm{rand}(\mathrm{VarSize})*(\mathrm{VarMax}-\mathrm{VarMin})+\mathrm{VarMin},\\ \mathrm{VelMin}=-0.1*\mathrm{VarMax}-\mathrm{VarMin},\\ \mathrm{VelMax}=-0.1*\mathrm{VarMax}-\mathrm{VarMin}. \end{array}\right.$$

Step 2: For each particle $X_{i}\left(t\right)$,

(e) A set of vehicle routes $K_{i}\left(t\right)$ is determined by decoding $X_{i}\left(t\right)$.
(f) The fitness value of $K_{i}\left(t\right)$ is determined by the objective function $\varphi \left(X_{i}\left(t\right)\right)$.
(g) The personal best position of particle *i* is identified as $P_{i}^{best}=X_{i}\left(t\right)$.
(h) The global best position $G^{best}$ of all particles is identified. If $\varphi \left(P_{i}^{best}\right)<\varphi \left(G^{best}\right)$, $G^{best}=P_{i}^{best}$. Otherwise, $G^{best}$ remains unchanged.

Step 3: For each iteration *it*,

(i) The velocity and position of particle *i* according to Equation (17) are updated. *N*(0,1) represents the standard normally distributed random numbers.

(17) $$\left\{ \begin{array}{c} \mu =w_{\min}+\left(w_{\max}-w_{\min}\right)*\mathrm{rand}\left(0,1\right),\\ w(t)=\mu +\sigma *N\left(0,1\right),\\ V_{i+1}(t+1)=w(t)V_{i}(t)+C_{1}R_{1}\;(P_{i}^{\mathrm{best}}\left(t\right)-X_{i}\left(t)\right)+C_{2}R_{2}(G^{\mathrm{best}}\left(t\right)-X_{i}\left(t\right)),\\ X_{i+1}(t+1)=X_{i}(t)+V_{i+1}(t+1),\\ \mathrm{if}\;V_{i+1}(t+1)>\mathrm{VelMax},\;V_{i+1}(t+1)=\mathrm{VelMax},\;\mathrm{if}V_{i+1}(t+1)<\mathrm{VelMin},\;V_{i+1}(t+1)=\mathrm{VelMin},\\ \mathrm{if}\;X_{i+1}(t+1)>\mathrm{VelMax},\;X_{i+1}(t+1)=\mathrm{VelMax},\\ \mathrm{if}\;X_{i+1}(t+1)<\mathrm{VelMin},\;X_{i+1}(t+1)=\mathrm{VelMin}. \end{array}\right.$$

(j) A set of vehicle routes $K_{i}$(*t*+1) is updated by decoding $X_{i}$(*t*+1).
(k) $P_{i}^{best}$: $P_{i}^{best}=X_{i}$(*t*+1) is updated, if $\varphi \left(X_{i}\left(t+1\right)\right)<\varphi \left(P_{i}^{best}\right)$.
(l) $G^{best}$: $G^{best}=P_{i}^{best}$ is updated, if $\varphi \left(P_{i}^{best}\right)<\varphi \left(G^{best}\right)$.
(m) When the number of iterations is greater than the number of population *nPop*, the current partial optimization solution $G^{best}$ calculated by the PSO is regarded as the initial solution of TS: *Y* = $G^{best}$.
(n) Three kinds of neighborhood search algorithms, swap, reversion, and insertion, are randomly selected to improve the partial optimization solution *Y*.
(o) The tabu list is renewed based on the special rules. Thus, the final selected solution is taken as the optimal solution $Y^{*}$.
(p) Return to step (i) until the maximum number of iteration *MaxIt* is met.
(q) $Y^{*}$ as the best set of vehicle routes $K^{*}$ is decoded, with its corresponding fitness value $\varphi \left(Y^{*}\right)$.

**Figure 2:**
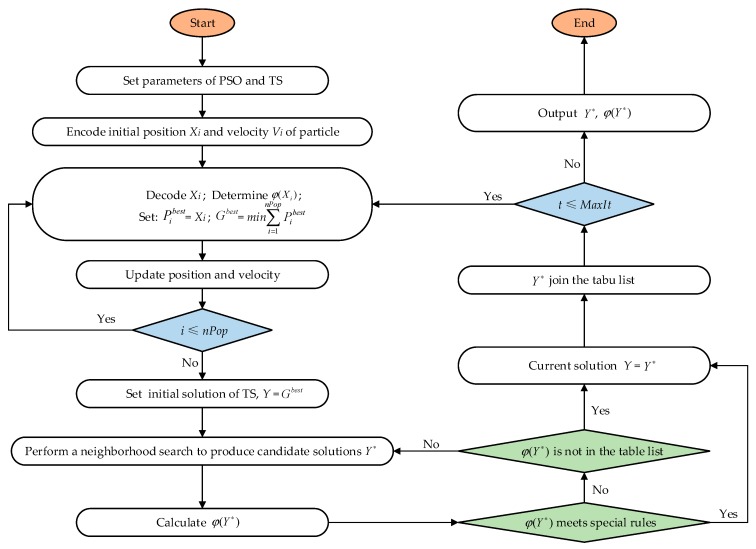
Basic procedures of the proposed particle swarm algorithm (PSO)-tabu search (TS) algorithm.

The operational flow of the proposed PSO-TS algorithm is described in Figure 2. 

### 4.2. Solution Representation and Decoding Method

The solution representation of CVRP with *N* collection points, 1 depot, *S* disposal facilities, and *M* sub-paths consists of 2*N*+*M+*1 dimensional particles. All particles are composed of four parts: encodings of sub-paths, collection points, disposal facilities, and the depot.

Sub-path encoding: Part 1 has *N* particles. The value of each particle represents the sub-path number to which each collection point belongs and is randomly selected from the natural number of 1 to $m\;\left(m=\overset{n}{\underset{i=1}{\sum }}q_{i}/Q\right)$. It is noted that *m* is the minimum number of sub-paths. 

Collection point encoding: Part 2 also has *N* particles. Its value represents the order of all collection points in each sub-path and is chosen from the natural number of 1 to *N* at random. There is one-to-one correlation in part 1 and 2, meaning that the collection points in part 2 corresponding to the particles having the same value in part 1 belong to the same path. 

However, while the sub-path number is calculated by the total demand dividing the vehicle capacity, the load of several sub-paths may also surpass the limit of load capacity. Thus, it is necessary to compute the load of each vehicle after firstly assigning the collection points to each sub-path. Counting the accumulated load of each sub-path by adding the demand of each point. If the load of the sub-path exceeds the limit, a new sub-path appears and the remaining collection points belongs to the new sub-path. Hence, the whole number of sub-path will be increased to a new number *M*.

Disposal facility encoding: Part 3 has *M* particles. The value of each particle represents the initial disposal facility corresponding to each sub-path and is stochastically selected from the natural number of *N*+1 to *N*+*S*;

Depot encoding: Part 4 has only one particle. Since all vehicles start from the same depot, we just encode the only one depot as the natural number of *N*+*S*+1.

Therefore, the total length of coding is 2*N*+*M*+1. 

For example, there are 10 collection points (number 1 to 10), 4 disposal facilities (number 11 to 14), and 1 depot (number 15). The sub-path number is *M* = 4 (number 1 to 4) which has been identified as the applicable number for not surpassing the vehicle capacity. A schematic illustration of the decoding example and the solution representation for the example problem are shown in Table 2 and Table 3. 

## 5. Experimental Design and Results Analysis

The proposed CVRP model and PSO-TS algorithm are verified by testing benchmark data with different sizes of collection points and disposal facilities. All simulation datasets utilized in this paper are derived from [43]. The experiment was conducted with Matlab R2016b on a computer with an Intel i5 @ 1.60 GHz Processor with 4 GB RAM. Based on three previous studies [18,44,45], the parameters of PSO-TS algorithm are set by adjusting the values so as to be suitable for this model, as shown in Table 4. 

### 5.1. Algorithm Experiment

The benchmark datasets of the multi-depot vehicle routing problem (MDVRP) are employed in this paper rather than the datasets of CVRP. Since all initial datasets of CVRP only have one depot location, Qu Wei et al. [46] used CVRP benchmark datasets and randomly selected a group of nodes to represent the disposal facilities. However, different locations of disposal facilities may bring about different routing and scheduling of vehicles. Thus, in order to diminish the impact of site selection for disposal facility, this paper adopts MDVRP datasets which have multiple appropriate locations of sites. Among all of these sites, the first site is identified as the only depot in this model, and the other sites are affirmed as disposal facilities for recycling waste.

As shown in Table 5, six instances without duration constraints of routes are chosen. The information of each instance contains the number of collection points, the depot, disposal facilities, vehicles, the capacity of vehicles, and workload limit U of each disposal facility (DF). The number of vehicles remains the same as in the initial instance. U, which means the upper limit of sub-paths for each DF, is set to the value of the number of vehicles which the initial depot owns. Besides, each DF has the same limit.

The optimization effectiveness of the proposed PSO-TS algorithm is tested by a comparison with the traditional PSO algorithm. Referring to Shen et al. [11] and Buhrkal et al. [47], each of the following experiments was carried out 10 times, and the best value was recorded as the optimal result.

Table 6 shows the computational results of PSO and PSO-TS including the number of sub-paths and distance. The optimization rate was also calculated to more clearly see the performance of PSO-TS. 

Obviously, compared with the results of the PSO, the number of sub-paths and distance calculated by PSO-TS are better. Thereby, the proposed PSO-TS algorithm has a great performance in improving the quality of solutions.

### 5.2. Model Experiment

#### 5.2.1. Experimental Design

Since the proposed algorithm is more applicable to small-scale instances [11], for which the number of collection points is not larger than 100, instance p01 is utilized to verify the proposed CVRP model. Similarly, each of the following experiments is performed 10 times and the best outcome is identified as the optimal result. 

The initial number of disposal facilities (DFs) in dataset p01 is too small for testing the effect of balancing the workload of DF (disposal facility). Hence, another three appropriate positions from collection points are chosen to be the new DFs. In this way, the numbers of DFs and collection points change from the original 3 and 50 to 6 and 47, respectively. The final determined positions of the six DFs is shown in Table 7. The information about the depot and vehicles is shown in Table 8, including the position of the depot, the number of vehicles, and the maximal load capacity of the vehicle. 

Referring to the method from Hannana et al. [18] and Jing-Quan Li et al. [13], the updated dataset p01 is scheduled for 5 days of one week to show the improvement in a realistic scenario. Waste loads for another 4 days are correlated with the original data of p01. Based on the primitive data, a change rate (θ), a changed mean value ($\bar{x}$), and a fixed standard deviation (σ) are considered for the waste quantity of each collection point. The change is normally distributed among all waste collection points. The change rate θ is set to vary at 15 percent intervals, that is, 15%, 30%, 45%, and 60%, and the mean value $\bar{x}$ is calculated by θ and original total quantity. The standard deviation σ is set as 0.25. The changed quantity is added to the original waste. Accordingly, the total waste for 6 days is 749, 976, 1197, 1426, and 1645 units, respectively. The information about positions and waste loads of collection points on Monday are presented in Table 9.

For the purpose of examining the effect of balancing, this paper conducts a contrast experiment (model 1) without balancing the number of sub-paths unloading the waste at DFs. The proposed CVRP model is denoted as model 2. This paper adopts a two-phase calculation, which means a set of feasible routes is obtained by model 1; then an optimized set of routes is found by model 2. 

In order to obtain the balancing results of model 2, different upper limits for each DF are set for the 5 days. Similarly to the literature [13], an integer is chosen as the given limit for each DF, which is greater or equal to the average number of sub-paths that is assigned to each DF. The proposed algorithm is then executed for a week (denoted as Monday, Tuesday, Wednesday, Thursday, and Friday). If a facility is assigned excessive collection trips, the given limit is decreased. Table 10 presents the upper limits for all 6 DFs, where $U_{r}$ represents the given limit for disposal facility *r*. 

According to the previous studies [10,11,42,48], the parameters related to the CVRP model are shown in Table 11. The penalty value *p* is set to half of the fixed costs per unit vehicle. 

#### 5.2.2. Experimental Results

To examine the impact of social equity on the vehicle routing, economic costs, and environmental benefits, we conducted two experiments: model 1 with minimized economic costs and carbon emissions, and model 2 with minimized economic costs, carbon emissions, and penalty costs of unbalanced workloads. Four selection criteria were used to compare the results of model 1 and model 2: the distance of routes, carbon emissions, operational costs, and sampling variance, respectively. Operational costs include fixed costs (the number of vehicles used), fuel consumption costs, and carbon emission costs. Penalty costs, which are utilized for balancing the workload of DFs (disposal facilities), were included in the fitness evaluation of model 2 but were not involved in model 1, because model 1 does not consider balancing the workload for DFs. Sampling variance (*SV*) is the variance of the number of sub-paths assigned to each DF (disposal facility). The value of *SV* is crucial to observe the effect of balancing. Thus, a balanced schedule with the smallest *SV* is preferred, even with slightly higher costs. 

**Figure 3:**
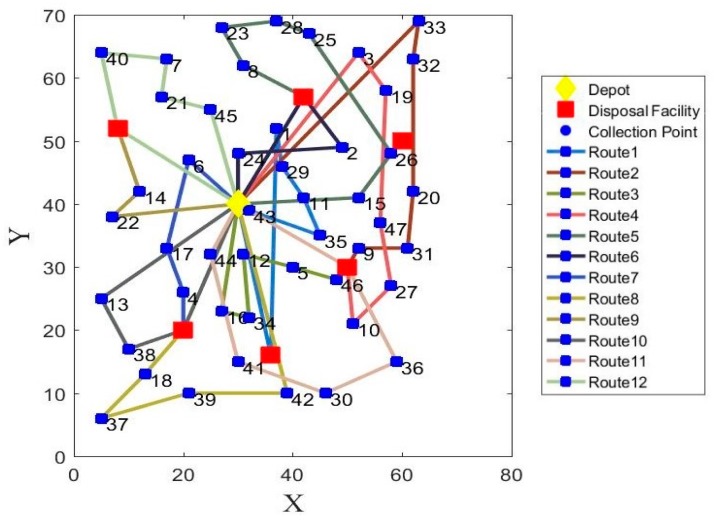
The optimal vehicle routes with minimized operational costs and carbon emissions on Monday (model 1).

**Figure 4:**
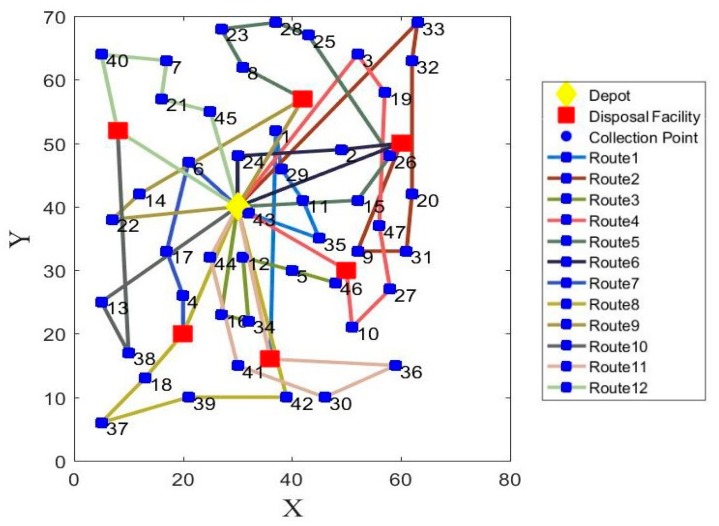
The optimal vehicle routes with minimized operational costs, carbon emissions, and penalty costs on Monday (model 2).

Since the model experiment is simulated for a week, taking the optimal solutions of Monday for example, the vehicle routes of model 1 and model 2 are shown in Figure 3 and Figure 4. The detailed results of model 1 and model 2 from Monday to Friday are separately presented in Table 12 and Table 13. The detailed collection routes of model 1 and model 2 from Monday to Friday are presented in Appendix A Table A2.

From the detailed results in Table 12 and Table 13, we can observe the following:

(1) When minimized penalty costs are added to the objective function in model 2, the values of *SV* obtained by model 2 are smaller than the values in model 1 every single day. Therefore, model 2 is efficient for improving social equity by acquiring balanced trip assignments of disposal facilities.
(2) After accumulating for a whole week, the *SV* is 2.97 in model 2, while the value is 55.97 in model 1. However, for each day, the values of *SV* are between 0 and 3.5 in the two models. Therefore, the imbalanced phenomenon can be more severe in the long-term in model 1.
(3) In the meantime, the distance, carbon emissions, and operational costs of model 2 all increase in the results of model 1. Thus, we infer that there is a trade-off between economic costs, environmental benefits, and social equity.

**Figure 5:**
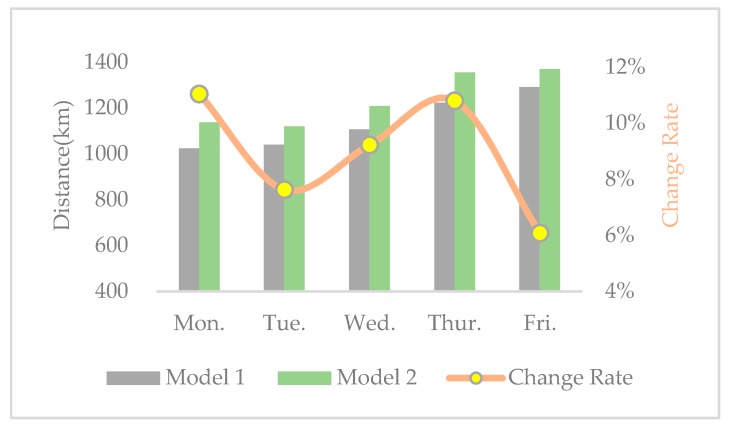
Distance and change rate in the two models.

**Figure 6:**
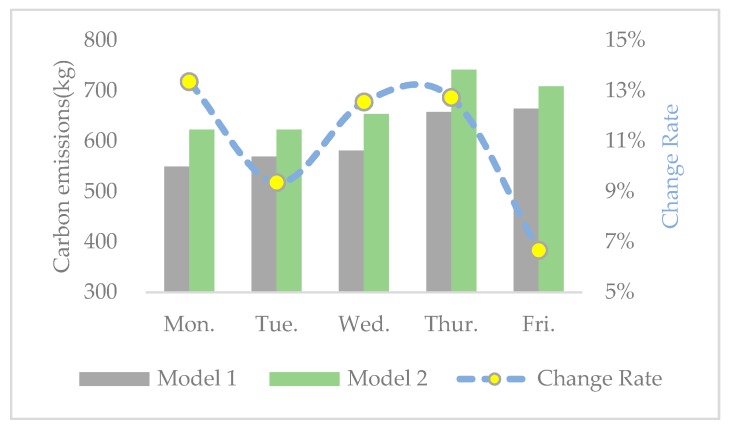
Carbon emissions and change rate in the two models.

**Figure 7:**
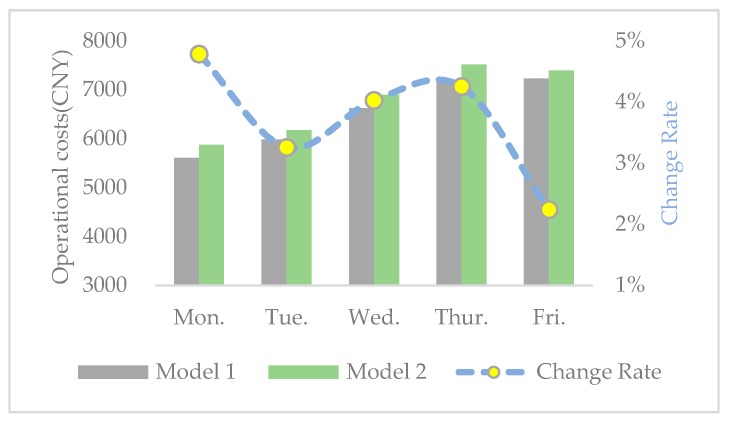
Operational costs and change rate in the two models.

**Figure 8:**
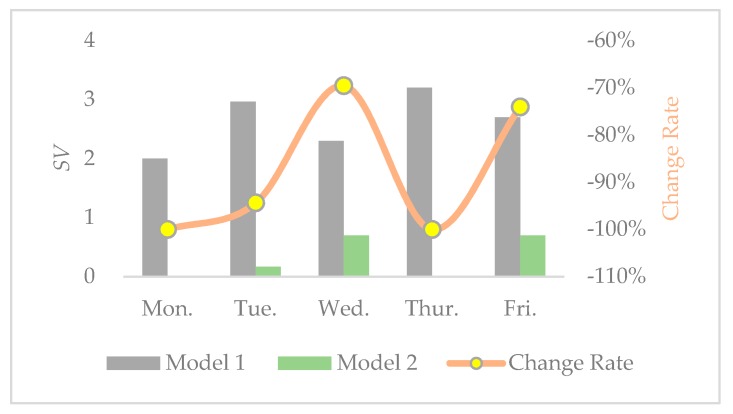
*SV* and change rate in the two models.

In order to further observe the interrelationship among economic, environmental, and social benefits, the change rates of distance, carbon emissions, operational costs, and SV from model 1 to model 2 are calculated and shown in Figure 5, Figure 6, Figure 7 and Figure 8, respectively. From the results in Figure 5, Figure 6, Figure 7 and Figure 8, we can observe the following findings:

(1) The change trends of distance, carbon emissions, and operational costs coincide every day, that is, they ascend and descend simultaneously at each turning point. Therefore, we infer that there is a positive correlation between economic and environmental benefits.
(2) The change rates of distance, carbon emissions, and operational costs are all situated in the interval between 1% and 15%, while the change rate of SV varies from −60% and −110%. Therefore, compared with the increase of social equity, the decrease in economic and environmental benefits is much smaller.

**Figure 9:**
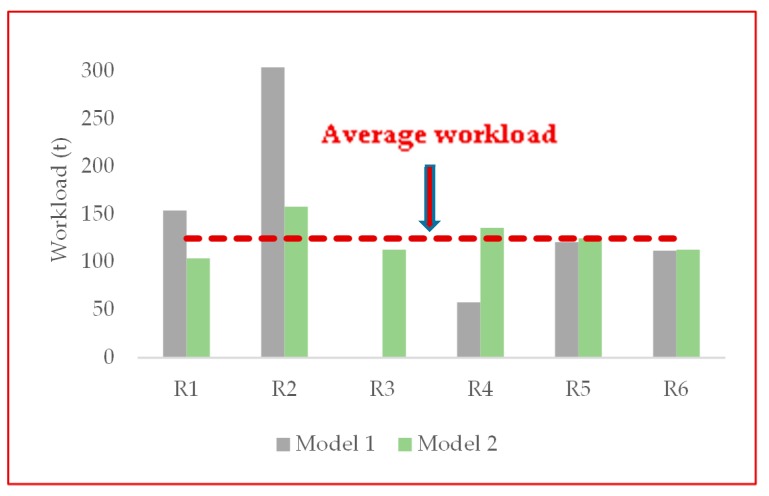
Workload of six disposal facilities in two models and the average workload on Monday (Average workload = 125 t).

In addition, in order to demonstrate that the balancing the trip assignments of DFs is effective for balancing the workload, we have taken the results of Monday as an illustration, as shown in Table 14 and Figure 9. From the value of workload in Table 14, in model 1, the difference between the maximum and the minimum workload is 304 t. However, this value in model 2 is 54 t, showing that the balancing method is effective to reduce the gaps in the workload. Figure 9 presents the discrepancy between each facility’s workload and the average workload, showing that the discrepancy is obviously narrowed in model 2. Thus, by balancing the trip assignments of DFs, the balanced workload of DFs can be obtained.

Overall, all the results imply that model 2 provides a sustainable solution for a good compromise among the operational costs, carbon emissions, and balanced workload for disposal facilities. In model 1, the distance and operational costs could be reduced, but imbalanced dispatches are generated.

### 5.3. Analysis of Results

For the vehicle routing and scheduling problem with multiple disposal facilities in municipal solid waste collection, the CVRP model is built in this paper to optimize economic, environmental, and social benefits simultaneously. By minimizing collection costs including vehicles’ fixed costs, fuel consumption costs, carbon emission costs, and penalty costs for unbalanced workload of disposal facilities, a comprehensive optimization collection and assigning scheme can be achieved. The main results of this paper are summarized as follows:

1. The proposed CVRP model can simultaneously take into account economic cost, environmental benefits (carbon emissions), and social equity (balanced workload of disposal facilities), resulting in a sustainable solution.
2. There is a certain trade-off between economic costs, environmental benefits, and social equity. Social equity can be increased between 60% and 110% when economic and environmental benefits only decrease between 1% and 15%.
3. There is a positive correlation between economic costs and environmental benefits, which can be combined into one objective.

Based on the above findings, some constructive suggestions are put forward.

For waste collection organizations, under the increased awareness of cost-effectiveness, environmental protection, and social equity perceptions, low-cost, low-carbon collection, and balanced assignments have been the key issues. Therefore, it is a wise choice to find a better compromise between these factors for the development of an organization. Firstly, an operational research method can be adopted to perform scientific collection routes so as to diminish collection costs. Secondly, introducing new energy transportation vehicles could reduce carbon emissions and save energy simultaneously. Thirdly, collection route assignments of disposal facilities can be balanced to equalize the workload of multiple facilities.

For governmental environmental protection, firstly, government departments should remain committed to the sustainable development of waste collection, not only improving economic and environmental benefits, but also considering social equity. Secondly, these departments could take into account formulating and issuing a series of policies to promote the efficiency and low-carbon transportation of waste collection, while guaranteeing the welfare of the staff in the recycling facilities.

## 6. Discussion

Within the background of sustainable development of MSW management, this paper utilizes a PSO-TS algorithm-based optimization model, which uses the analysis of the interrelationship among the economic costs, environmental benefits, and social equity from the perspective of the MSW collection process. Referring to the balancing method from proposed by Li et al. [13], and incorporating vehicle capacity and carbon emissions as aspects which Li et al. [13] did not include, we found a comprehensive sustainable solution could be obtained by considering economic, environmental, and social benefits together. 

Compared with the existing studies on the sustainable development of waste management, this paper has contributed some unique findings. Although the evaluation criteria of social concern are different, as social equity was defined as the same number of driving hours by Pereira Ramos et al. [6], a compromise among the economic, environmental, and social benefits has been obtained. For the relationship between economic costs and environmental benefits, Pereira Ramos et al. [6] found there are only slight trade-offs between them, and we observed that they have a positive relationship when the social equity is added to the optimization model. Why do economic and environmental benefits show a similar variation tendency when they are considered together with social equity? We suppose that the main reason is that more reasonable and cost-effective collection route assignments are beneficial for reducing economical costs and carbon emissions. In addition, another significant finding is that a small decrease in economic and environmental benefits could result in a great increase in social equity. This discovery is helpful for promoting the policies and schemes of social equity so as to guarantee the welfare of human resources. 

However, there are also limitations of the study and the established model, showing directions for further research. Firstly, this paper focuses on a holistic perspective and the waste is considered as a whole for collection. However, effective waste classification is helpful for disposal, and collection systems in Japan, the United States, and many European countries are based on the collection of several waste types [20,49,50]. In China, waste classification is also being gradually promoted. The General Office of the State Council released the *Implementation Plan for Domestic Garbage Classification System* in 2017 [50] and 46 cities have been approved as pilot cities. Therefore, within the frame of future research, we are going to focus on the different types of waste and study the waste-integrated collection scheme. In addition, technology has shown its advantages for the convenient management of waste collection, for example GIS, which can record the real-time traffic data of vehicles, and smart waste bins which can monitor waste status [17,18]. Such intelligent technologies should also be considered in future research.

## 7. Conclusions

The problem of planning a sustainable MSW collection system taking into account economic costs, environmental benefits, and social equity has been studied. The results of numerical experiments suggest that the compromise between economic costs, environmental benefits, and social equity is worthwhile. The scientific contribution of this paper for researchers in this field is the mathematical modelling and optimization of MSW collection processes based on a hybrid algorithm composed of particle swarm optimization and tabu search. The results can be generalized, since the model and optimization method can be applied for the collection of different kinds of waste such as medical waste and kitchen waste.

## Figures and Tables

**Table 1:** Notations of the proposed capacitated vehicle routing problem (CVRP) model.

Variables	Explanation
$x_{\mathrm{ijh}}$	$x_{\mathrm{ijh}}=1$, if vehicle h visits from point i to point j, Otherwise, $x_{\mathrm{ijh}}=0$
$y_{\mathrm{ih}}$	$y_{\mathrm{ih}}=1,$ if vehicle h visits point i, Otherwise, $y_{\mathrm{ih}}=0$
$z_{\mathrm{mhi}}^{r}$	$z_{\mathrm{mhi}}^{r}=1,$ if sub-path m of vehicle h unloads waste at disposal facility r, includes point i served by the vehicle h, Otherwise, $z_{\mathrm{mhi}}^{r}=0$
$f_{m}^{r}$	$f_{m}^{r}=1,$ if sub-path m assigns to disposal facility r causing overload of facility r, Otherwise, $f_{m}^{r}=0$
**Parameters**	**Explanation**
G	Set of all the nodes in the graph network, G={V, K}
K	Set of vehicles {h|h=1, 2, …, H}
V	Set of collection points {i|i=0, 1, 2, …, N}, 0 is the depot
R	Set of disposal facilities {r|r=1, 2, …,S}
T	Set of sub-paths {m|m=1, 2, …,M}
$Q_{\mathrm{ijh}}$	Carried load of vehicle h visit from point i to point j
Q	Maximal load capacity of the vehicle
$q_{i}$	Waste collection demand of collection point i
$U_{r}$	Workload limit of disposal facility r
$d_{\mathrm{ij}}$	Transportation distance from point i to point j
$c_{v}$	Fixed costs of per unit vehicle
$c_{f}$	Cost of per unit fuel consumption
$c_{e}$	Cost of per unit carbon emission
η	Fuel consumption rate when vehicle is full-loadConsumption Rate
$\eta_{0}$	Fuel consumption rate when vehicle is empty
λ	Conversion factor for carbon dioxide and fuel consumption
p	Penalty cost of overload disposal facility for per sub-path

**Table 2:** Schematic illustration of the decoding example.

**Part 1**	1	1	3	2	3	2	1	1	4	2
**Part 2**	3	6	5	1	8	4	2	10	7	9
**Part 3**	11	13	11	12						
**Part 4**	15									

**Table 3:** Solution representation for the example problem.

Vehicle Routes	
1	15-3-6-2-10-11-15
2	15-1-4-9-13-15
3	15-5-8-11-15
4	15-7-12-15

**Table 4:** Parameters connected to the particle swarm optimization (PSO)-tabu search (TS) algorithm.

Parameters of the PSO	Values	Parameters of the TS	Values
MaxIt	1000	TL	20
nPop	50	NS	$C_{Varsize}^{2}$
w_max_	0.8	CS	(0.1*$C_{Varsize}^{2}$)
w_min_	0.5		
σ	0.2		
R_1_, R_2_	rand (Varsize)		
C_1_, C_2_	1.5		
VarMin	0		
VarMax	1		

**Table 5:** Data of the test instances.

Instance	Collection Point	Depot	Disposal Facility	Workload Limit	Vehicle	Capacity
p01	50	1	3	4	16	80
p02	50	1	3	2	8	160
p03	70	1	4	3	15	140
p06	100	1	2	6	18	100
p07	100	1	3	4	16	100
p15	160	1	3	5	20	60

**Table 6:** Computational results of the PSO and PSO-TS.

Instance	PSO		PSO-TS		Optimization Rate (%)
	Number of Sub-Paths	Distance	Number of Sub-Paths	Distance	
p01	14	1517.24	12	1175.85	22.50%
p02	6	1183.91	6	904.23	23.62%
p03	13	1874.15	12	1369.59	26.92%
p06	19	2940.19	18	2445.96	16.81%
p07	18	2701.45	18	2196.23	18.70%
p15	18	14,376.75	17	11,528.92	19.81%
Average	-	-	-	-	21.39%

**Table 7:** Positions of all disposal facilities.

Disposal Facilities	X Coordinate	Y Coordinate
1	20	20
2	50	30
3	60	50
4	36	16
5	42	57
6	8	52

**Table 8:** Position of the depot and information about vehicles.

Depot	X Coordinate	Y Coordinate	Number of Vehicles	Maximal Weight/t
1	30	40	16	80

**Table 9:** Positions and waste load of collection points on Monday.

**Collection Points**	**1**	**2**	**3**	**4**	**5**	**6**	**7**	**8**	**9**	**10**	**11**	**12**
X	37	49	52	20	40	21	17	31	52	51	42	31
Y	52	49	64	26	30	47	63	62	33	21	41	32
Waste Load/t	7	30	16	9	21	15	19	23	11	5	19	29
**Collection Points**	**13**	**14**	**15**	**16**	**17**	**18**	**19**	**20**	**21**	**22**	**23**	**24**
X	5	12	52	27	17	13	57	62	16	7	27	30
Y	25	42	41	23	33	13	58	42	57	38	68	48
Waste Load/t	23	21	15	3	41	9	28	8	16	28	7	15
**Collection Points**	**25**	**26**	**27**	**28**	**29**	**30**	**31**	**32**	**33**	**34**	**35**	**36**
X	43	58	58	37	38	46	61	62	63	32	45	59
Y	67	48	27	69	46	10	33	63	69	22	35	15
Waste Load/t	14	6	19	11	12	23	26	17	6	9	15	14
**Collection Points**	**37**	**38**	**39**	**40**	**41**	**42**	**43**	**44**	**45**	**46**	**47**	
X	5	10	21	5	30	39	32	25	25	48	56	
Y	6	17	10	64	15	10	39	32	55	28	37	
Waste Load/t	7	27	13	11	16	10	5	25	17	18	10	

**Table 10:** Different upper limits for disposal facilities.

**Day**	**Upper Limits for the Number of Sub-Paths**					
	**$U_{1}$**	**$U_{2}$**	**$U_{3}$**	**$U_{4}$**	**$U_{5}$**	**$U_{6}$**
Monday	2	2	2	2	2	2
Tuesday	2	2	2	2	2	2
Wednesday	3	3	3	3	3	3
Thursday	3	3	3	3	3	3
Friday	4	4	4	4	4	4

**Table 11:** Parameters of the proposed CVRP model.

Parameters	Values
$c_{v}$	300 CNY (Chinese Yuan)
$c_{f}$	7 CNY/L
$c_{e}$	0.64 CNY/kg
η	0.377 L/km
$\eta_{0}$	0.165 L/km
λ	2.32 kg/L
p	150 CNY

**Table 12:** Results of model 1: Minimized operational costs and carbon emissions.

Day	Sub-Paths	Distance (km)	Carbon Emissions (kg)	Operational Costs (CNY)	Sub-Path Assignments of Disposal Facilities						SV
					$R_{1}\;$	$R_{2}\;$	$R_{3}\;$	$R_{4}\;$	$R_{5}\;$	$R_{6}\;$	
Monday	12	1023.50	351.46	5608.39	3	4	0	1	2	2	2.00
Tuesday	13	1039.31	364.43	5982.53	4	3	0	1	4	1	2.97
Wednesday	15	1105.62	371.89	6625.14	4	4	0	2	3	2	2.30
Thursday	18	1222.04	421.18	7206.82	4	5	0	2	3	4	3.20
Friday	21	1290.33	425.17	7229.59	5	5	2	1	4	4	2.70
Week	79	5680.80	1934.13	32,652.48	20	21	2	7	16	13	55.77

**Table 13:** Results of model 2: Minimized operational costs, carbon emissions, and penalty costs.

Day	Sub-Paths	Distance (km)	Carbon Emissions (kg)	Operational Costs (CNY)	Sub-Path Assignments of Disposal Facilities						SV
					$R_{1}$	$R_{2}$	$R_{3}$	$R_{4}$	$R_{5}$	$R_{6}$	
Monday	12	1136.56	398.41	5876.69	2	2	2	2	2	2	0.00
Tuesday	13	1118.65	398.53	6177.38	2	3	2	2	2	2	0.17
Wednesday	15	1207.62	418.57	6891.89	3	3	1	2	3	3	0.70
Thursday	18	1354.04	474.80	7513.22	3	3	3	3	3	3	0.00
Friday	21	1368.75	453.50	7391.49	4	4	3	2	4	4	0.70
Week	79	6185.62	2143.81	33,850.68	14	15	11	11	14	14	2.97

**Table 14:** Detailed route assignments and workload of disposal facilities on Monday in the two models.

Model	Detailed Route Assignments of Disposal Facilities on Monday					
	$R_{1}$	$R_{2}$	$R_{3}$	$R_{4}$	$R_{5}$	$R_{6}$
Model 1	6-17-4-$R_{1}$ 42-39-37-18-$R_{1}$ 13-38-$R_{1}$	33-32-20-31-9-$R_{2}$ 16-34-12-5-46-$R_{2}$ 3-19-47-27-10-$R_{2}$ 44-41-30-36-$R_{2}$	--	43-35-11-29-1-$R_{4}$	15-26-25-28-23-8-$R_{5}$ 24-2-$R_{5}$	22-14-$R_{6}$ 45-21-7-40-$R_{6}$
Workload/t	154	304	0	58	121	112
Model 2	6-17-4-$R_{1}$ 42-39-37-18-$R_{1}$	16-34-12-5-46-$R_{2}$ 3-19-47-27-10-$R_{2}$	33-32-20-31-9-$R_{3}$ 24-2-$R_{3}$	43-35-11-29-1-$R_{4}$ 44-41-30-36-$R_{4}$	15-26-25-28-23-8-$R_{5}$ 22-14-$R_{5}$	13-38-$R_{6}$ 45-21-7-40-$R_{6}$
Workload/t	104	158	113	136	125	113

## Appendix A

**Table A1:** Notations and parameters of the PSO-TS algorithm.

PSO Parameters	Explanation
t	Iteration index: t=1, 2, …, MaxIt
i	Population index: i=1, 2, …, nPop
VarSize	Length of particle code
w(t)	Random inertia weight in the itth iteration
w_max_	Maximum value of inertia weight
w_min_	Minimum value of inertia weight
σ	Variance of random inertia weight
R_1_	Random number in the interval [0, 1]
R_2_	Random number in the interval [0, 1]
C_1_	Personal acceleration factor
C_2_	Global acceleration factor
VarMin	Lower bound of the position for each particle
VarMax	Upper bound of the position for each particle
VelMin	Lower bound of the velocity for each particle
VelMax	Upper bound of the velocity for each particle
Vi(t)	Velocity of particle i in the tth iteration
Xi(t)	Position of particle i in the tth iteration
Ki(t)	Set of vehicle routes corresponding to particle i in the tth iteration
$P_{i}^{best}$	Personal best position of particle i
$G^{best}$	Global best position of all particles
$\varphi \left(X_{i}\left(t\right)\right)$	Fitness value of Xi(t)
TS Parameters	Explanation
TL	Tabu length
NS	Neighborhood size
CS	Candidate size

**Table A2:** Detailed collection routes of model 1 and model 2 from Monday to Friday.

Day	Model 1	Model 2
Monday	6-17-4-$R_{1}$, 42-39-37-18-$R_{1}$, 13-38-$R_{1}$, 33-32-20-31-9-$R_{2}$, 16-34-12-5-46-$R_{2}$, 3-19-47-27-10-$R_{2}$, 44-41-30-36-$R_{2}$, 43-35-11-29-1-$R_{4}$, 15-26-25-28-23-8-$R_{5}$, 24-2-$R_{5}$, 22-14-$R_{6}$, 45-21-7-40-$R_{6}$	6-17-4-$R_{1}$, 42-39-37-18-$R_{1}$, 16-34-12-5-46-$R_{2}$, 3-19-47-27-10-$R_{2}$, 33-32-20-31-9-$R_{3}$, 24-2-$R_{3}$, 43-35-11-29-1-$R_{4}$, 44-41-30-36-$R_{4}$, 15-26-25-28-23-8-$R_{5}$, 22-14-$R_{5}$, 13-38-$R_{6}$, 45-21-7-40-$R_{6}$
Tuesday	16-41-39-37-18-$R_{1}$, 44-17-$R_{1}$, 13-38-$R_{1}$, 14-22-4-$R_{1}$, 1-34-10-36-27-9-$R_{2}$, 5-46-$R_{2}$, 33-32-26-20-31-$R_{2}$, 12-42-30-$R_{4}$, 43-11-29-3-25-$R_{5}$, 6-45-8-28-$R_{5}$, 19-$R_{5}$, 35-47-15-2-$R_{5}$, 24-23-7-21-40-$R_{6}$	44-17-$R_{1}$, 13-38-$R_{1}$, 5-46-$R_{2}$, 1-34-10-36-27-9-$R_{2}$, 33-32-26-20-31-$R_{2}$, 6-45-8-28-$R_{3}$, 19-$R_{3}$, 12-42-30-$R_{4}$, 14-22-4-$R_{4}$, 35-47-15-2-$R_{5}$, 43-11-29-3-25-$R_{5}$, 16-41-39-37-18-$R_{6}$, 24-23-7-21-40-$R_{6}$
Wednesday	22-13-18-$R_{1}$, 39-37-38-$R_{1}$, 12-34-16-4-$R_{1}$, 44-17-$R_{1}$, 35-15-47-$R_{2}$, 20-31-27-$R_{2}$, 36-10-46-9-$R_{2}$, 5-$R_{2}$, 30-42-41-$R_{4}$, 43-11-29-1-$R_{4}$, 32-33-3-25-$R_{5}$, 19-26-2-$R_{5}$, 24-8-23-28-$R_{5}$, 14-6-21-$R_{6}$, 45-7-40-$R_{6}$	5-$R_{1}$, 22-13-18-$R_{1}$, 12-34-16-4-$R_{1}$, 20-31-27-$R_{2}$, 35-15-47-$R_{2}$, 36-10-46-9-$R_{2}$, 24-8-23-28-$R_{3}$, 39-37-38-$R_{4}$, 30-42-41-$R_{4}$, 32-33-3-25-$R_{5}$, 14-6-21-$R_{5}$, 19-26-2-$R_{5}$, 45-7-40-$R_{6}$, 43-11-29-1-$R_{6}$, 44-17-$R_{6}$
Thursday	4-$R_{1}$, 41-39-18-$R_{1}$, 44-17-$R_{1}$, 13-37-38-$R_{1}$, 36-10-31-$R_{2}$, 15-27-$R_{2}$,12-16-46-$R_{2}$, 11-35-9-$R_{2}$, 5-47-26-20-$R_{2}$, 2-1-$R_{4}$, 43-34-30-42-$R_{4}$, 29-3-25-$R_{5}$, 45-23-28-$R_{5}$, 19-32-33-$R_{5}$, 6-$R_{6}$, 24-8-21-$R_{6}$, 7-40-$R_{6}$, 22-14-$R_{6}$	22-14-$R_{1}$, 41-39-18-$R_{1}$, 4-$R_{1}$, 12-16-46-$R_{2}$, 11-35-9-$R_{2}$, 36-10-31-$R_{2}$, 19-32-33-$R_{3}$, 45-23-28-$R_{3}$, 5-47-26-20-$R_{3}$, 43-34-30-42-$R_{4}$, 15-27-$R_{4}$, 2-1-$R_{4}$, 7-40-$R_{5}$, 6-$R_{5}$, 29-3-25-$R_{5}$, 44-17-$R_{6}$, 13-37-38-$R_{6}$, 24-8-21-$R_{6}$
Friday	39-41-34-16-$R_{1}$, 4-12-$R_{1}$, 13-38-$R_{1}$, 37-18-$R_{1}$, 17-$R_{1}$, 10-46-5-$R_{2}$, 30-42-36-$R_{2}$, 27-$R_{2}$, 47-31-9-$R_{2}$, 11-29-35-$R_{2}$, 15-26-$R_{3}$, 32-33-3-$R_{3}$, 43-24-1-$R_{4}$, 2-$R_{5}$, 25-28-$R_{5}$, 45-23-8-$R_{5}$, 20-19-$R_{5}$, 40-$R_{6}$,44-6-$R_{6}$, 22-14-$R_{6}$, 21- 7-$R_{6}$	39-41-34-16-$R_{1}$, 17-$R_{1}$, 13-38-$R_{1}$, 37-18-$R_{1}$, 10-46-5-$R_{2}$, 30-42-36-$R_{2}$, 27-$R_{2}$, 47-31-9-$R_{2}$, 20-19-$R_{3}$, 15-26-$R_{3}$, 32-33-3-$R_{3}$, 43-24-1-$R_{4}$, 4-12-$R_{4}$, 2-$R_{5}$, 25-28-$R_{5}$, 11-29-35-$R_{5}$, 40-$R_{5}$, 44-6-$R_{6}$, 22-14-$R_{6}$, 21-7-$R_{6}$, 45-23-8-$R_{6}$
